# MicroRNA-7 mediates cross-talk between metabolic signaling pathways in the liver

**DOI:** 10.1038/s41598-017-18529-x

**Published:** 2018-01-10

**Authors:** Ragunath Singaravelu, Curtis Quan, Megan H. Powdrill, Tyler A. Shaw, Prashanth Srinivasan, Rodney K. Lyn, Rhea C. Alonzi, Daniel M. Jones, Roxana Filip, Rodney S. Russell, John P. Pezacki

**Affiliations:** 1 Department of Biochemistry, Microbiology and Immunology, University of Ottawa, Ottawa, Ontario, K1N 6N5 Canada; 2 Department of Chemistry and Biomolecular Sciences, University of Ottawa, Ottawa, Ontario, K1N 6N5 Canada; 3 Immunology and Infectious Diseases, Faculty of Medicine, Memorial University of Newfoundland, St. John’s, Newfoundland, A1B 3V6 Canada

## Abstract

MicroRNAs (miRNAs) have emerged as critical regulators of cellular metabolism. To characterise miRNAs crucial to the maintenance of hepatic lipid homeostasis, we examined the overlap between the miRNA signature associated with inhibition of peroxisome proliferator activated receptor-α (PPAR-α) signaling, a pathway regulating fatty acid metabolism, and the miRNA profile associated with 25-hydroxycholesterol treatment, an oxysterol regulator of sterol regulatory element binding protein (SREBP) and liver X receptor (LXR) signaling. Using this strategy, we identified microRNA-7 (miR-7) as a PPAR-α regulated miRNA, which activates SREBP signaling and promotes hepatocellular lipid accumulation. This is mediated, in part, by suppression of the negative regulator of SREBP signaling: ERLIN2. miR-7 also regulates genes associated with PPAR signaling and sterol metabolism, including liver X receptor β (LXR-β), a transcriptional regulator of sterol synthesis, efflux, and excretion. Collectively, our findings highlight miR-7 as a novel mediator of cross-talk between PPAR, SREBP, and LXR signaling pathways in the liver.

## Introduction

The human liver plays a central role in systemic metabolism^1^. Proper regulation of gene networks in the liver is integral to the maintenance of energy homeostasis^1^. Several transcription factors have been established as key regulators of lipid and lipoprotein metabolism in the liver, including sterol response element binding proteins (SREBPs), liver X receptors (LXRs), and peroxisome proliferator activated receptors (PPARs)^2–5^. PPARs are a family of nuclear hormone receptors which function as transcription factors for genes associated with lipid metabolism and inflammation^2^. In the liver, PPAR-α is the most highly expressed PPAR isoform, and regulates fatty acid catabolism and lipid export^3^. Similarly, SREBP1C is the most highly expressed SREBP isoform in the adult liver and, this family of transcription factors regulates genes associated with lipid biosynthesis^4,5^. Two isoforms of LXR exist (α and β), and both regulate sterol synthesis, efflux and excretion in the liver^6^. As these transcription factors exert profound effects on overlapping aspects of hepatic metabolism, significant cross-talk is required between these signaling pathways to coordinate lipid homeostasis.

Several studies have examined the interplay between these signaling pathways^7–11^; however, the majority have focused on coding genes. It is well established that LXRs and PPARs heterodimerize with a common partner, retinoid X receptor (RXR), to mediate their transcriptional effects^7,8^. Additionally, LXRs are known to directly transcriptionally activate SREBP1C expression^12^. Therefore, PPARs and LXRs compete for RXR binding to activate their respective signaling pathways, and PPAR-α overexpression interferes with LXR-mediated activation of SREBP1 expression^7,8^. These studies suggest competition between the LXR and PPAR signaling pathways; however, an independent study reported that PPAR-α and LXR share genomic binding sites^9^. In fact, it was demonstrated that PPAR-α can bind the LXR response element in the promoter of SREBP1C to mediate transcriptional activation^11^. Collectively, these results point to complex interplay between PPAR-α, LXR, and SREBP signaling. To-date, the majority of studies have focused on the role of coding genes in this complex cross-talk. Characterisation of non-coding RNAs that are co-regulated by these metabolic pathways could help explain the underlying complexities of this cross-talk.

Recent work has illustrated that microRNAs (miRNAs) act as an important regulatory layer in the control of hepatic metabolism^13^. These 21–24 nucleotide, small, non-coding RNAs repress gene expression post-transcriptionally through partial pairing with mRNAs, yielding a combination of translational repression and mRNA destabilisation^14^. The importance of miRNAs in metabolic controls is supported by observations of aberrant hepatic miRNA profiles in metabolic disorders, including diabetes/insulin resistance^15–17^, obesity^18^, non-alcoholic fatty liver disease^19^, and hepatitis C virus (HCV)-associated steatosis^20–22^.

In the current study, we sought to characterise miRNAs regulating PPAR, LXR, and SREBP signaling to gain insight into the molecular mechanisms of cross-talk between these metabolic pathways. Our findings suggest a novel role for a PPAR-α regulated miRNA, miRNA-7 (miR-7), in the regulation of SREBP signaling. miR-7 stimulates the activity of SREBPs, master regulators of lipid biosynthesis. We demonstrate that miR-7-dependent activation of triglyceride synthesis and lipid storage is mediated, in part, through inhibition of ERLIN2, a negative regulator of SREBP signaling. miR-7 appears to further regulate lipid homeostasis through downregulation of LXR-β expression. Furthermore, genome-wide expression profiling reveals that miR-7 overexpression modulates the expression of several genes associated with cholesterol and fatty acid metabolic processes. Collectively, our work highlights miR-7 as a novel mediator of cross-talk between the PPAR-α, LXR-β, and SREBP signaling pathways.

## Results

### PPAR-α signaling regulates miR-7 expression

Chronic HCV infection is associated with a high prevalence of hepatic steatosis. The development of steatosis is linked to the virus’ perturbations of SREBP, LXR, and PPAR signaling. *In vitro* studies have demonstrated that HCV viral proteins promote SREBP maturation^23,24^ and LXR gene expression and transcriptional activity^25^. Impaired PPAR-α expression was observed during HCV infection, both *in vitro* and *in vivo* ^26^. Therefore, we utilised HCV infection as a model to identify the influence of miRNAs on these liver metabolism-regulating pathways.

**Figure 1:**
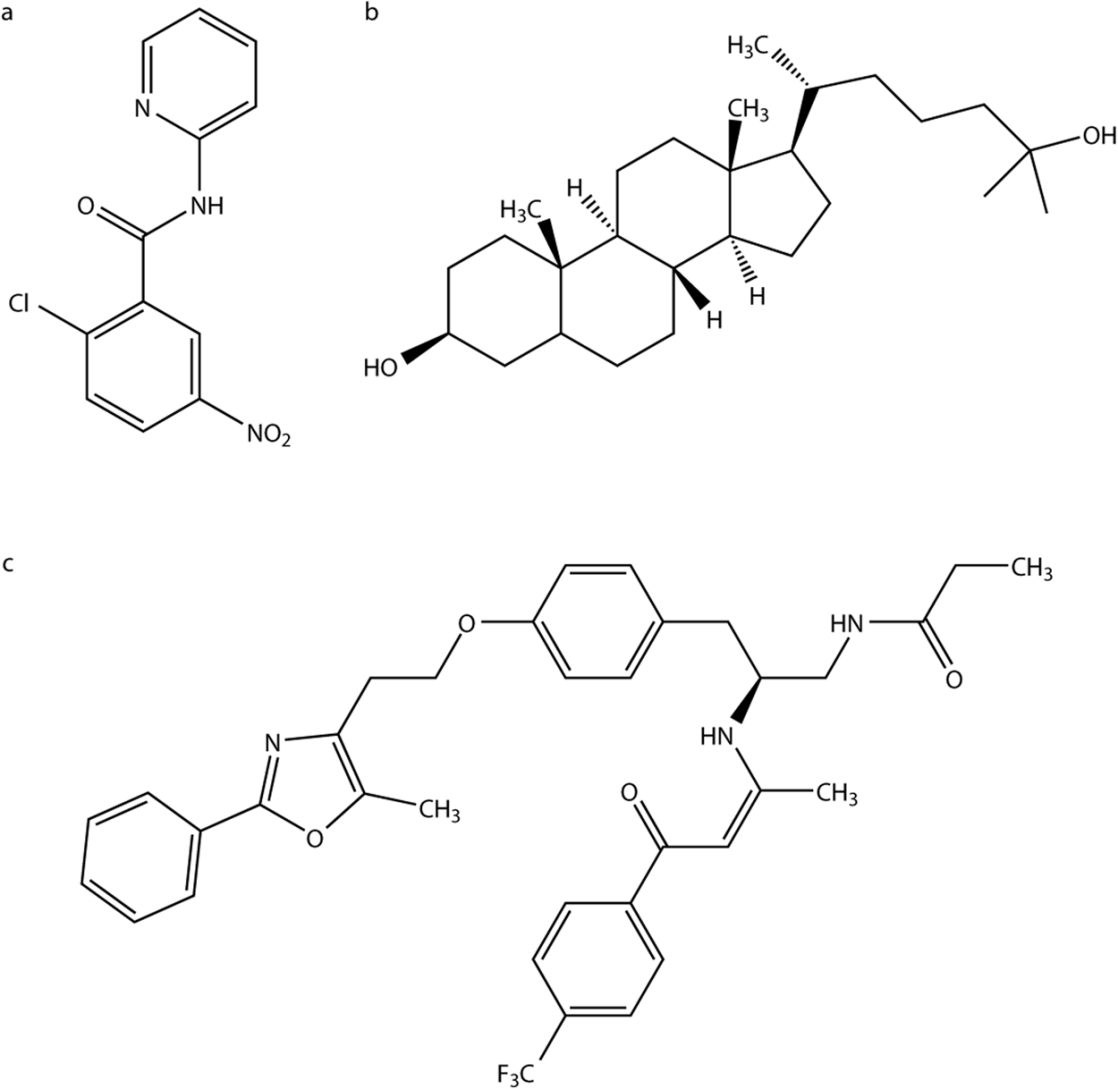
Structures of the compounds used in this study. Molecular structures of the PPAR-α antagonist 2-chloro-5-nitro-N-(pyridyl)benzamide (**a**), the LXR agonist 25-HC (**b**), and the PPAR-α antagonist GW6471 (**c**).

**Figure 2:**
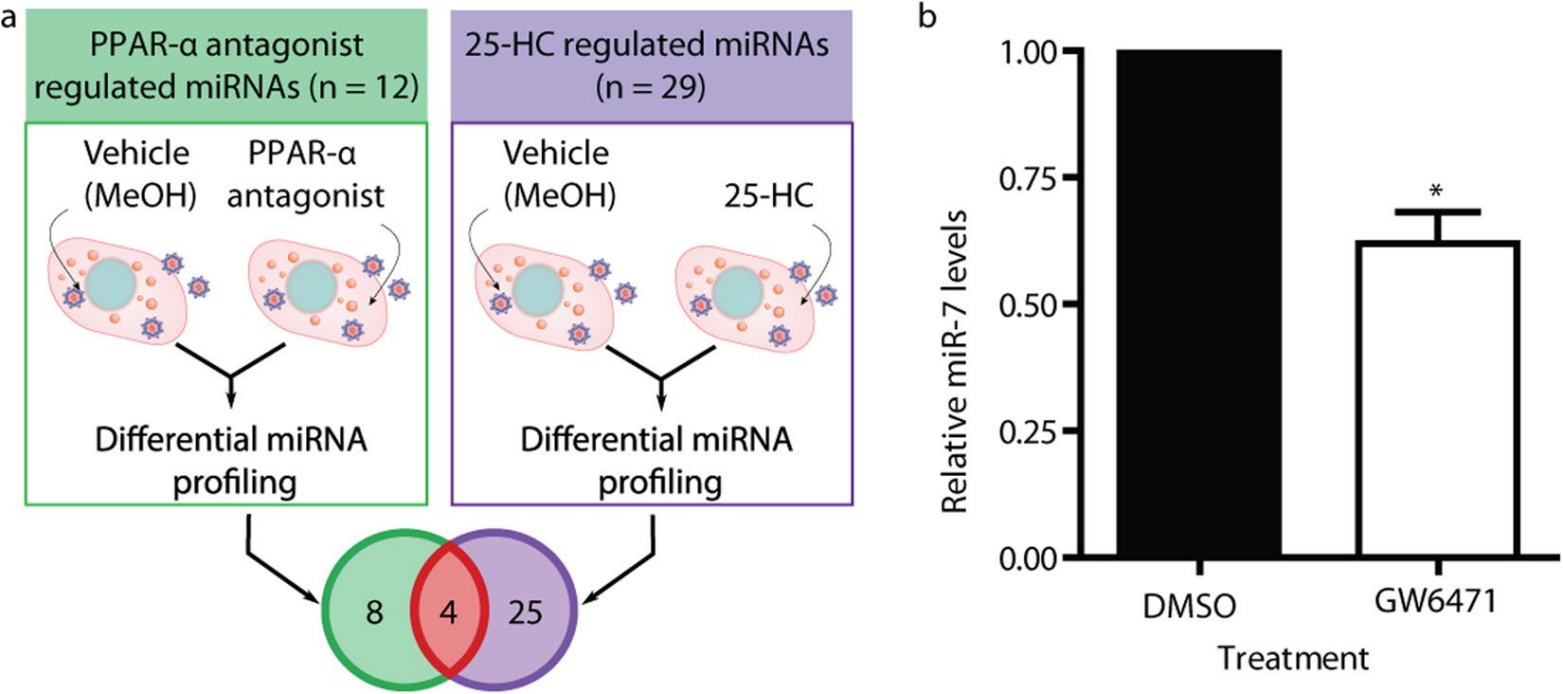
MicroRNA-7 regulates cross-talk between metabolic signaling pathways. (**a**) Overview of the miRNA profiling strategy applied to identify miRNAs regulating cross-talk between metabolic signaling pathways. (**b**) Huh7.5 cells were treated with 10 μM of the PPAR-α antagonist GW6471 for 24 h. qRT-PCR was performed to measure relative miR-7 expression (n = 4). An unpaired two-tailed t-test was used to evaluate statistical significance (*P ≤ 0.05).

To identify miRNAs regulating cross-talk between metabolic signaling pathways, we performed miRNA microarray profiling in HCV-infected Huh7.5 hepatoma cells treated with a PPAR antagonist, 2-chloro-5-nitro-N-(pyridyl)benzamide (BA)^27,28^ (Fig. 1a). We compared this miRNA signature to our previously reported list of miRNA candidates regulated by 25-hydroxycholesterol (25-HC; Fig. 1b), an inhibitor of SREBP maturation and agonist of the LXR pathway^29^, in HCV-infected cells^22^. We hypothesised that miRNAs regulated by both PPAR-α and 25-HC (Fig. 2a) were likely to play regulatory roles in multiple signaling pathways.

Twelve miRNAs are differentially expressed during PPAR-α antagonist treatment (Table 1). Of the four miRNAs differentially expressed during both 25-HC and PPAR-α antagonism (Table 2), miR-7-5p (miR-7) was selected for further study, as previous work has shown that miR-7 expression is regulated by HNF4α, a liver enriched transcription factor^30^, suggesting it plays a physiological role in the liver. The microarray data revealed repression of miR-7 expression by both small molecule treatments (Table 2). To confirm PPAR-α regulation of miR-7 expression, we treated uninfected Huh7.5 cells with GW6471 (Fig. 1c), a potent PPAR-α antagonist^31^, to examine miRNA expression in the absence of virus. qRT-PCR analysis revealed a 40% decrease in miR-7 levels (Fig. 2b), validating that PPAR-α signaling represses mature miR-7 abundance.

**Table 1:** miRNAs differentially expressed in HCV-infected Huh7.5 cells treated with a PPAR-α antagonist.

miRNA*	*P* value	Fold Change
hsa-miR-1244	1E-2	−2.78
hsa-miR-1255b-5p	1E-3	−6.67
hsa-miR-1304-5p	4E-2	−2.94
hsa-miR-183-5p	1E-3	−2.22
hsa-miR-302b-3p	3E-2	1.76
hsa-miR-509-5p	3E-2	−4.00
hsa-miR-516a-5p	4E-2	−2.00
hsa-miR-620	2E-2	−1.92
hsa-miR-647	5E-2	−3.23
hsa-miR-7-5p	2E-2	−6.25
hsa-miR-7-2-3p	5E-3	−2.86
hsa-miR-920	3E-2	−2.70

*Table includes miRNAs modulated at least 1.5-fold following 25 µM BA treatment of HCV-infected Huh7.5 cells (*P ≤ *0.05).

**Table 2:** miRNAs modulated by both a PPAR-α antagonist and 25-HC.

miRNA*	25-HC-induced differential miRNA expression	PPAR-α antagonist-induced differential miRNA expression
hsa-miR-1244	−3.62	−2.78
hsa-miR-509-5p	−9.35	−4.00
hsa-miR-647	−1.59	−3.23
hsa-miR-7-5p	−4.93	−6.25

*Table includes miRNAs modulated at least 1.5-fold during both 25 µM BA treatment and 5 μM 25-HC treatment of HCV-infected Huh7.5 cells (*P* ≤ 0.05).

### miR-7 stimulates SREBP signaling

**Figure 3:**
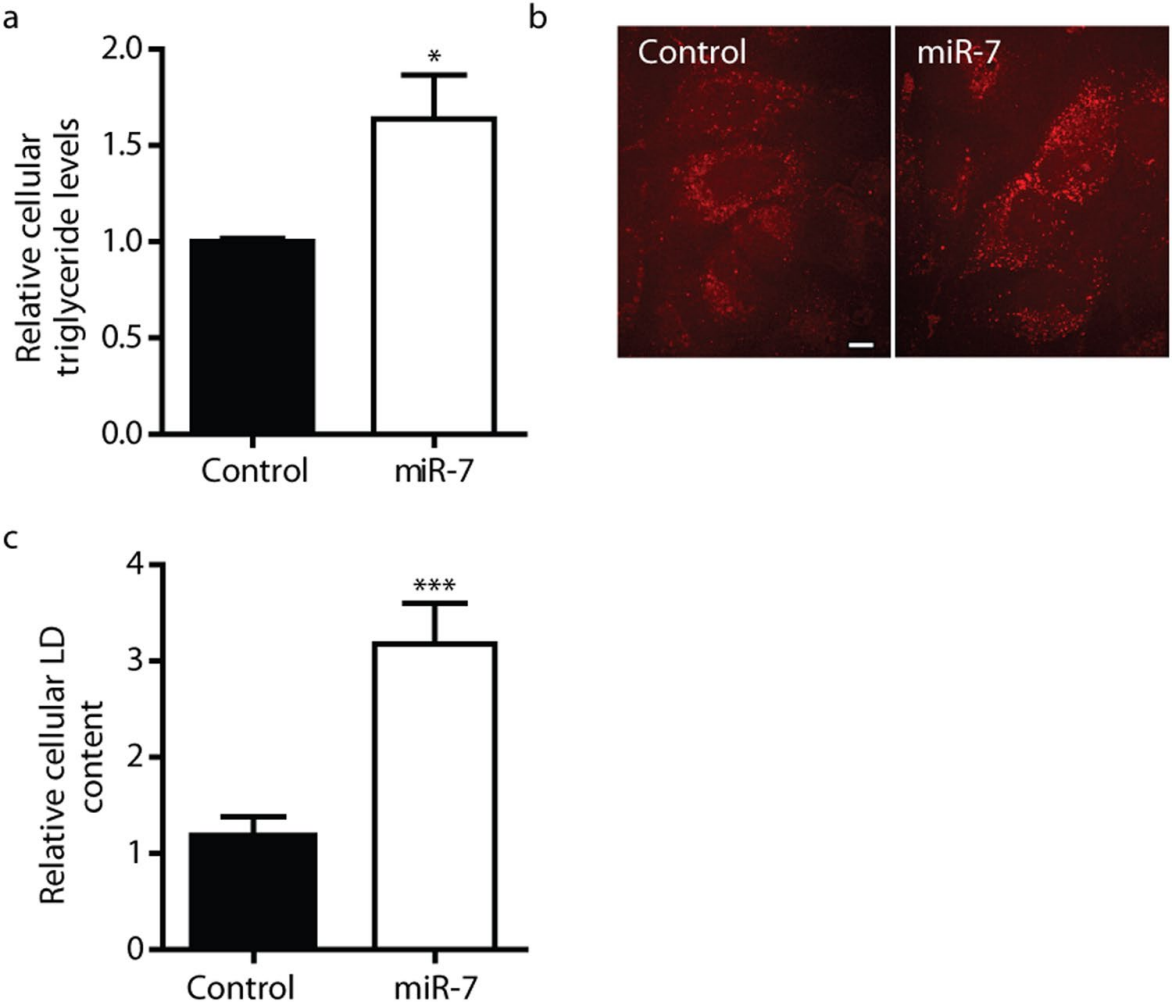
miR-7 promotes cellular lipid accumulation. (**a**) Relative cellular TG content in control and miR-7 mimic-transfected Huh7.5 cells as assessed by TG assays. (**b**) Representative CARS microscopy images of intracellular LDs in control and miR-7 mimic transfected Huh7 cells. Cells were fixed 48 h post-transfection. Scale bar represents 10 µm. (**c**) Quantitative analysis of relative total cellular LD content (n > 30 cells). Data represents mean values ± s.e.m. An unpaired two-tailed t-test was used to evaluate statistical significance (*P ≤ 0.05; ***P ≤ 0.001).

Since miR-7 expression levels are responsive to fatty acid levels in mouse myoblasts^32^, and the miRNA has previously been implicated in insulin signaling^33^, we hypothesised that miR-7 may play a role in hepatic metabolism. To gain insight into the role of miR-7 in metabolic pathways, we performed gene expression profiling of Huh7.5 cells transfected with miR-7 synthetic mimics (Supplementary Table S1). Bioinformatic analysis, using the ToppGene Suite^34^, was performed to identify potential transcription factors with binding sites enriched in the promoters of miR-7 activated genes. Interestingly, only SREBP1 binding sites were overrepresented in the promoters of genes upregulated greater than 1.5-fold during miR-7 overexpression (*P* < 1 × 10^−4^). These results suggest that miR-7 activates SREBP1 signaling. SREBP1c is considered a master transcriptional regulator of fatty acid and triglyceride (TG) synthesis^4^. We therefore predicted that miR-7-mediated activation of SREBP1 signaling should result in increased triglyceride levels. As anticipated, overexpression of miRNA-7 in Huh7.5 cells resulted in cellular TG accumulation, consistent with SREBP1 signaling activation (Fig. 3a).

### miR-7 promotes hepatocellular lipid accumulation

**Figure 4:**
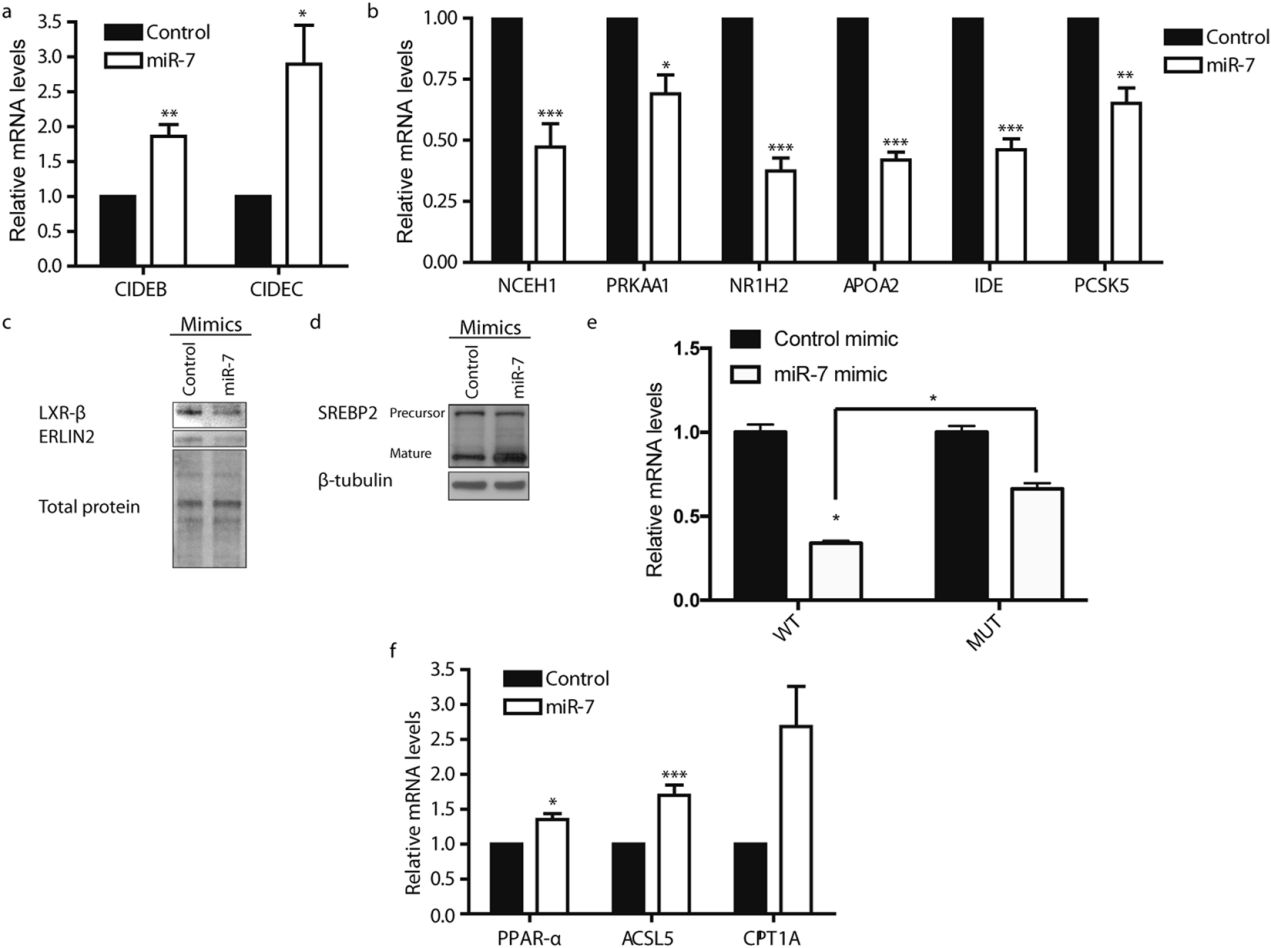
miR-7 regulates genes associated with PPAR signaling and hepatic lipid metabolism. Relative mRNA expression levels of (**a**) CIDEB and CIDEC and (**b**) miR-7 predicted targets (n = 4). (**c**) Immunoblot analysis of protein expression levels of LXR-β and ERLIN2 in cells treated with a control mimic or miR-7 mimic. Total protein detection serves as loading control. (**d**) Immunoblot analysis of protein expression levels of mature and precursor SREBP2 levels in cells treated with a control or miR-7 mimic. β-tubulin is shown as a loading control. Full uncropped blots are shown in Supplementary Fig. S5. (**e**) Relative luciferase activity in HEK293 cells transfected with bicistronic vectors encoding firefly luciferase reporter bearing either the NR1H2 3′UTR with either wildtype (WT) or mutated (PM) miR-7 binding sites and either a control or miR-7 mimic. Results are normalized relative to respective control mimic sample. (**f**) Relative mRNA expression levels of PPAR-α and PPAR-α targets. Data represents mean values ± s.e.m. An unpaired two-tailed t-test was used to evaluate statistical significance (*P ≤ 0.05; **P ≤ 0.01; ***P ≤ 0.001).

We subsequently used coherent anti-Stokes Raman scattering (CARS) microscopy to perform label-free analysis of the influence of miR-7 on hepatocellular lipid droplet (LD) content in Huh7 cells^35,36^. Our CARS analysis revealed that miR-7 promoted cellular LD accumulation (Fig. 3b,c), and an increase in the average diameter of visualised LDs from 600 ± 10 nm to 650 ± 10 nm (n > 9, 700 LDs; *P* < 0.05), consistent with the observed increase in cellular TG levels (Fig. 3a). As LD proteins have been implicated in the pathophysiology of hepatic steatosis^37^, we postulated that the miR-7-mediated changes in LD morphology of Huh7.5 cells may, in part, result from changes in the LD-associated proteome. We examined the genes which were differentially expressing during miR-7 overexpression for LD-localised proteins. The cell death inducing DFF45-like effector (CIDE) family of proteins are a family of LD-associated proteins which regulate LD clustering and fusion^38,39^. Notably, the microarray data revealed an increase in the expression of two members of the CIDE family, CIDEB and CIDEC (see Supplementary Fig. S1). We validated miR-7 mediated stimulation of CIDEB and CIDEC expression in Huh7.5 cells via qRT-PCR (Fig. 4a). Both CIDEB and CIDEC are known to induce LD clustering and fusion^38–40^, and increased CIDEC expression is correlated with steatosis^41,42^. Therefore miR-7-mediated activation of CIDEB and CIDEC expression likely contributes to the observed accumulation of larger LDs.

### miR-7 modulates expression of lipid metabolism-associated genes

Next, we sought to identify direct targets of miR-7, which could explain the increased SREBP1 signaling and hepatocellular lipid accumulation. We examined the overlap between miR-7 predicted targets from TargetScan^43^ and targets repressed by miR-7 by at least 1.5-fold. This list of 683 genes was then examined for negative regulators of SREBP1 signaling. Among the target candidates are two known inhibitors of SREBP maturation, ERLIN2 and PRKAA1, which encodes the α1 catalytic subunit of AMPK^44,45^ (Supplementary Figs S1 and S2). qRT-PCR demonstrated that miR-7 overexpression modestly downregulates PRKAA1 expression (Fig. 4b). Parallel immunoblot analyses revealed that miR-7 represses ERLIN2 (Fig. 4c); however, there was no significant decrease in AMPK α1 protein levels (Supplementary Fig. S3). ERLIN2 promotes retention of SREBPs at the endoplasmic reticulum, preventing the proteolysis required for its activation^44^. Consistent with this, we observed increased processing of SREBP2 into its mature form during miR-7 overexpression (Fig. 4d). Furthermore, qRT-PCR confirmed an increase in the expression of SREBP-regulated genes (SQLE, SCD1, and FASN) (Supplementary Fig. S4). Therefore, miR-7 repression of ERLIN2 expression contributes to miR-7 stimulated SREBP signaling.

Using the Panther classification system^46^, we examined the list of repressed miR-7 putative targets to identify additional genes functionally associated to metabolism. This analysis produced NR1H2, APOA2, PCSK5, IDE, and NCEH1 as additional repressed miR-7 targets of interest (Supplementary Figs S1 and S2). qRT-PCR validated miR-7-mediated decrease of NR1H2, APOA2, IDE, and NCEH1 mRNA levels by greater than 50% (Fig. 4b). NR1H2 encodes LXR-β, suggesting that PPAR-α activation of miR-7 expression contributes to suppression of LXR-β signaling. Therefore, we examined protein expression of NR1H2 in miR-7 or control mimic-treated Huh7.5 cells and confirmed a decrease in NR1H2 levels (Fig. 4c). To validate NR1H2 as a direct target of miR-7, we performed a 3′ untranslated region (UTR) luciferase reporter assay which showed decreased activity in the presence of the miR-7 mimic (Fig. 4e). This inhibition was alleviated when the predicted miR-7 binding site was mutated, confirming direct interaction of miR-7 with the NR1H2 3′UTR. These results demonstrate that miR-7 mediates concerted regulation of several genes with functional associations to hepatic metabolism.

In order to further classify biological processes activated by miR-7, we performed gene ontology analysis on genes upregulated by more than 1.5 fold in miR-7 transfected Huh7.5 cells^34^. The list of statistically significant activated mRNA transcripts shows an overrepresentation of genes involved in lipid catabolism and sterol metabolism (Supplementary Table S2). Gene set enrichment analysis (GSEA) was also performed on the miR-7 and control mimic-transfected Huh7.5 gene expression profiles to identify additional pathways modulated by miR-7. Interestingly, the top pathways enriched in genes positively correlated with miR-7 expression included PPAR signaling (Supplementary Table S3). qRT-PCR analysis revealed a modest increase in PPAR-α expression levels (Fig. 4f); however, no significant change in protein levels were observed (Supplementary Fig. S3). On the other hand, we observed significant increases in the expression of known PPAR-α targets, including ACSL5^47^ (Fig. 4f) and CIDEC^41^ (Fig. 4a). Taken together, these results suggest that miR-7 stimulates PPAR-α signaling through a mechanism independent of influencing PPAR-α abundance.

## Discussion

To date, miRNAs have been shown to play regulatory roles in different aspects of hepatic lipid metabolism^13,48^. As post-transcriptional regulators of gene expression, miRNAs add a level of functional complexity to classical regulatory gene networks. In this study, we sought to identify miRNAs regulating the cross-talk between important metabolic signaling pathways in the liver. By applying small molecule modulators of three important transcription factors (PPAR, LXR, and SREBP) in conjunction with miRNA profiling, we uncovered a novel role for miR-7 as a PPAR-α regulated miRNA that inhibits LXR-β and activates SREBP1 signaling. Our work re-emphasises the utility of small molecules for identifying miRNAs regulating specific host pathways^22^.

The observed miRNA signature for PPAR-α antagonism included other miRNAs with known links to lipid metabolism, including miR-183 and miR-302b (Table 1). miR-183 is transcribed as part of as a conserved polycistronic cluster of miRNAs^49^, which includes miR-182 and miR-96. Interestingly, previous work demonstrated that expression of miRNAs derived from this cluster was regulated by PPAR signaling^50,51^, consistent with our profiling results. miR-183 has been implicated in SREBP activation^52^ and insulin signaling^53^ while miR-302 has been shown to regulate cholesterol efflux^54^. Collectively, these data suggest that the PPAR-α regulated miRNAs play an important role in regulating hepatic metabolism.

miR-7 is evolutionarily conserved across bilateral species^55^, suggesting it plays an functional role. Previous work examining miR-7 function in the liver ascribed the miRNA a role in tumor suppression^56^. Our miRNA profiling demonstrated that both 25-HC, a LXR agonist and inhibitor of SREBP signaling, and PPAR-α antagonist treatment down-regulated miR-7 expression levels. Regulation by two different metabolic inhibitors highlights a potential role for the miRNA in hepatic lipid pathways. qRT-PCR analysis validated mature miR-7 as a PPAR-α-regulated miRNA; however, in humans, miR-7 expression derives from three separate loci in the genome (MIR7-1, MIR7-2, and MIR7-3). Our miRNA microarray data also demonstrated a downregulation in miR-7-2-3p levels. Since miR-7-5p and miR-7-2-3p can derive from the same miRNA precursor, our data suggests MIR7-2 is the genomic locus at which PPAR-α regulation is occurring.

Our results point to a lipogenic role for miR-7 in the liver as miR-7 overexpression results in cellular LD and TG accumulation. This steatotic phenotype is consistent with the observed miR-7-induced gene expression profile, as inhibition of ERLIN2 promotes lipid accumulation and SREBP1 signaling^2,44^. In addition to negative regulation of SREBP1, ERLIN2 mediates degradation of HMGCR, an enzyme catalyzing the rate limiting step of cholesterol biosynthesis^57^. Furthermore, we also observed upregulation of CIDEC, whose expression correlates with hepatic steatosis^41,42^. Overall, our results suggest that the lipogenic function of miR-7 results from the cooperative effect of regulating multiple metabolism-associated genes. While our data points to miR-7 repression of ERLIN2 resulting in increased cholesterol and triglyceride synthesis, we cannot exclude the possibility that miR-7 is also influencing lipid export and catabolism to mediate the observed increase in cellular lipid content, as well there are potentially other unidentified miR-7 targets contributing to these effects.

**Figure 5:**
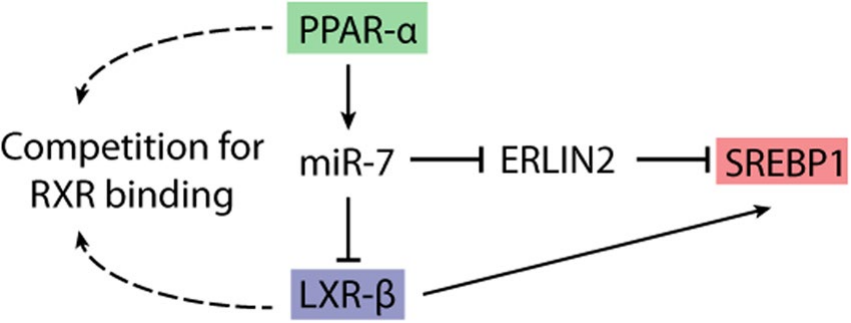
Proposed model of miR-7’s role in cross-talk between PPAR, SREBP, and LXR signaling pathways. PPAR-α positively regulates miR-7 expression. miR-7 represses the expression of putative targets, LXR-β and ERLIN2. PPAR-α competes with LXR-β for binding to RXR to mediate its transcriptional effects. PPAR-α-mediated activation of miR-7 expression further suppresses LXR signaling, through direct targeting of LXR-β. While PPAR-α inhibits LXR-mediated SREBP1 transcriptional activation, stimulating miR-7 expression appears to rheostat this effect through suppression of a negative regulator of SREBP1 activity (ERLIN2).

Our study also revealed miR-7 acts as a novel mediator of cross-talk between the PPAR-α, LXR-β, and SREBP1 signaling pathways (Fig. 5). PPAR-α and LXR compete for RXR binding to mediate their transcriptional effects^58,59^. As PPAR-α mediated activation of miR-7 expression represses LXR-β expression, increased miR-7 levels should promote PPAR-α signaling by decreasing the LXR-mediated competition for RXR binding^7,8^. Therefore, our proposed PPAR-α-miR-7-LXR-β signaling axis can account for miR-7’s stimulatory effect on PPAR signaling. Furthermore, LXR transcriptionally activates SREBP1 expression^12^, so PPAR-α mediated suppression of LXR signaling should result in decreased SREBP signaling. However, our work suggests that PPAR-α rheostats its inhibitory effect on the SREBP1 pathway by promoting miR-7-mediated stimulation of SREBP1 maturation.

miR-7-mediated repression of APOA2 and IDE also points to potential roles for the miRNA in high-density lipoprotein (HDL) biogenesis and hormone secretion. APOA2 encodes apolipoprotein II-A, which is the second most abundant protein on HDLs^60^, suggesting that miR-7-mediated regulation of APOA2 expression could impact HDL biogenesis. Previous work has highlighted an important role for miR-7 in glucose-stimulated insulin secretion from the pancreas^61^. IDE, or insulin degrading enzyme, encodes an enzyme responsible for catabolism of glucagon, amylin, and insulin^62,63^. Thus, our work suggests miR-7 induced repression of IDE expression could also contribute to miR-7’s regulatory effects on hormones secreted from the pancreas.

In summary, our data suggest a functional role for miR-7 in hepatic lipid homeostasis at the intersection of PPAR, SREBP, and LXR signaling pathways. This PPAR-α-regulated miRNA regulates SREBP1 signaling, hepatocellular lipid accumulation, and cholesterol metabolism through the concerted regulation of ERLIN2 and LXR-β. This evolutionarily conserved miRNA plays a lipogenic role in the liver, and our study opens new avenues for exploration of miR-7’s regulatory effects in metabolism in the context of other tissues and systemic energy homeostasis.

## Experimental Procedures

### Materials

The Huh7.5 cell line was a kind gift from Dr. Charles M. Rice (Rockefeller University, New York, NY) and Apath (St. Louis, MO)^64^. All *mir*Vana miRNA mimics and inhibitors, along with controls, were purchased from Ambion (Austin, TX). 2-chloro-5-nitro-N-(pyridyl)benzamide (BA; >98%) was purchased from Cayman Chemical Company (Ann Arbor, MI, USA). GW6471 (≥98%) was obtained from Sigma-Aldrich.

### Cell culture and transfections

Adherent Huh7.5 and Huh7 cells were cultured in Dulbecco’s Modified Eagle Medium (DMEM; Invitrogen, Burlington, ON) supplemented with 100 nM nonessential amino acids (NEAA; Gibco, Burlington,ON), 50 U/mL penicillin, 50 mg/mL streptomycin, and 10% fetal bovine serum (FBS; PAA Laboratories, Etobicoke, ON). Transfections were performed using Lipofectamine RNAiMax (Life Technologies, Carlsbad, CA) for miRNA mimics and inhibitors (Ambion). Transfections were performed according to the manufacturer’s protocol. GW6471 and DMSO (vehicle) treatments were performed for 24 h.

### HCV infection and miRNA microarray analysis

The HCV JFH-1_T_ strain possesses 3 amino acid changes that enhance infectious virus production and was derived from the cell culture-adapted JFH-1 strain JFH-AM1^65^. Twenty-four hours prior to infection, Huh-7.5 cells were seeded into 6-well plates. On the following day, cells were inoculated with infectious HCV particles (MOI = 0.1) for 4 h before the medium was removed and replaced with fresh media. For BA treatments, infected cells were treated 48 h post-infection with methanol (vehicle) or varying concentrations of BA. Twenty-four h post drug treatment (72 h post infection), cells were lysed with TriZol (Life Technologies) for RNA isolation. RNA integrity was verified by 0.8% agarose gel electrophoresis. Total RNA (3 μg) was labeled using the Oyster-550 based Flashtag RNA labelling kit (Genisphere). miRCURY LNA microRNA Array probe spotting, microarray hybridization and wash conditions, data extraction and image analysis were performed as previously described^22^. Quantile normalization was performed using the preprocessCore library of the Bioconductor package in the R environment. Each array was performed in at least triplicate from three biological replicates. Comparisons of BA-induced miRNA changes were made with our previously published miRNA profiles for methanol (vehicle) and 25-hydroxycholesterol (25-HC) treatment^22^, available at Gene Expression Omnibus (GSE73164).

### CARS microscopy

Huh7 cells were seeded in 4.2 cm^2^ Lab-Tek Chambers Slide System (NUNC). Prior to imaging, cells were washed twice with phosphate-buffered saline (PBS), and fixed in a 4% formaldehyde, 4% sucrose solution for 15 min at room temperature. Fixed cells were subsequently washed twice with PBS for 3 min and stored at 4 °C in PBS prior to imaging. The imaging, quantitative voxel analysis of cellular LD content, and LD sizing was performed using ImageJ (NIH), as previously described^27,38^.

### Triglyceride assay

Cellular triglyceride (TG) levels were analyzed directly by spectrophotometric analyses, using the BioVision TG quantification kit according to the manufacturer’s instructions. TG levels were normalized to total protein levels, which were quantified using the Bio-Rad DC protein assay.

### mRNA microarray analysis

RNA isolation from Huh7.5 cells was performed with the RNeasy kit (Qiagen). Gene expression profiling was performed using Affymetrix Human Gene ST.2.0 arrays. Data was normalized and analysed using the Affymetrix Expression Console and Transcriptome Analysis Console (v3.0), according to the manufacturer’s protocols. Gene ontology, pathway enrichment, and transcription factor binding site analysis was performed using the ToppGene Suite^34^ or Gene Set Enrichment Analysis (GSEA)^66^. For ToppGene analysis, *P* values were adjusted with Bonferroni correction. For GSEA analysis, bi-weight average signals (log2) from Affymetrix arrays were used as input. The KEGG pathway database was selected for target gene sets, and default settings were used with the exception of the metric for ranking genes (Ratio of Classes) and permutation type (Gene Set).

### Quantitative RT-PCR

RNA isolation from hepatocytes was performed using TriZol (Life Technologies), RNeasy kits (Qiagen), or NucleoSpin miRNA (Macherey-Nagel), according to the manufacturer’s protocol. RNA integrity was confirmed by 0.8% agarose gel electrophoresis in 1X TBE (Ambion). For mRNA profiling, 10 ng of total RNA was reverse transcribed using the Superscript II RT kit (Life Technologies) following the manufacturer’s instructions. qPCR was subsequently performed on an iCycler (Bio-Rad) using iQ SYBR Green SSO Advanced Supermix (Bio-Rad), according to the manufacturer’s protocol. Primer sequences are listed in Supplementary Table S4. Relative miRNA levels were quantified using the Taqman miRNA Assay (Applied Biosystems), with 10 ng of total RNA used for reverse transcription using the TaqMan MicroRNA Reverse Transcription Kit (Applied Biosystems). For both mRNA and miRNA quantification, the 2^−ΔΔCt^ method was used to calculate fold changes in expression relative to mock or control treated samples^67^, with 18 S rRNA or RNU6B levels being used for normalisation.

### Immunoblot analysis

Transfected cells were washed twice in PBS and lysed in a buffer containing 50 mM Tris-HCl (pH 6.8), 2% SDS and 10% glycerol. cOmplete Protease Inhibitor Cocktail tablets (Roche) were added to the lysis buffer. A DC Protein assay (Bio-Rad) was performed for quantification of protein and 40 µg of protein was resolved by SDS-PAGE. TGX Stain-Free gels where total protein loading is shown. Protein samples were transferred to a Hybond-P PVDF membrane (GE Healthcare Life Sciences). The membrane was then probed with either mouse anti-NR1H2 (1:500 dilution; Perseus Proteomics), rabbit anti-ERLIN2 (1:500 dilution, Cell Signaling Tech., 2959), mouse anti-SREBP2 (1:200 dilution, BD Pharmingen), rabbit anti-β-tubulin (1:2000, Santa Cruz Biotech., sc-9104), or rabbit anti-PPAR- α (1:500 dilution, Santa Cruz Biotech., sc-9000), or goat anti-AMPKα1 (1:200; Santa Cruz Biotech., sc-19128) followed by an appropriated secondary HRP-conjugated antibody; either goat anti-mouse antibody (1:20000; Jackson ImmunoResearch Laboratories, Inc.), donkey anti-goat antibody (1:10000, Jackson ImmunoResearch), or donkey anti-rabbit antibody (1:10000, Jackson ImmunoResearch). Blots were visualised with Clarity ECL Western blotting reagents (Bio-Rad). Gel loading controls were performed by either probing for β-tubulin or using stain-free detection of total protein loading (Bio-Rad). Blot images were cropped and contrast was adjusted using ImageJ (NIH) or Image Lab (Bio-Rad). Full images of blots are shown in Supplementary Fig. S5.

### 3′UTR luciferase reporter assay

miR-7 binding sites were mutated in the dual luciferase reporters bearing the 3′UTR of NR1H2 (Genecopoeia) using QuikChange Lightning kit (Stratagene), as per the manufacturer’s protocols, using the primers provided in Supplementary Table S4. HEK293 cells were seeded in 48-well plates, and transfected with wild-type and mutant luciferase reporter constructs. 24 hr post-transfection, cells were transfected with miR-7 or control mimics (100 nM). 48 hr post-transfection, cells were lysed in 1X passive lysis buffer (Promega). Luciferases assay were performed as previously described.

### Statistical analysis

Data is presented as the mean of replicates, with error bars representing the standard error of the mean. Unless otherwise stated, statistical significance was evaluated using an unpaired Student’s t-test, and *P-*values less than 0.05 were deemed significant.

### Accession numbers

Gene expression profiling data from miR-7 and control mimic transfected Huh7.5 cells and miRNA profiling data from PPAR-α antagonist treated HCV infected Huh7.5 cells have been deposited to NCBI Gene Expression Omnibus under the following accession numbers: GEO: GSE108267, GSE108268.

## Electronic supplementary material

supporting information
