# Supplementary material for: MicroRNA-7 mediates cross-talk between metabolic signaling pathways in the liver
Source: Sci Rep. 2018 Jan 10;8:361. doi: 10.1038/s41598-017-18529-x (PMC5762714; doi:10.1038/s41598-017-18529-x)
Supplement: Supplementary file 1 — supporting information [file 41598_2017_18529_MOESM1_ESM.pdf]

Supplementary Information

MicroRNA-7 mediates cross-talk between metabolic signaling pathways in the liver

Ragunath Singaravelu[1], Curtis Quan[1], Megan H. Powdrill[2], Tyler A. Shaw[2], Prashanth Srinivasan[1], Rodney K. Lyn[2], Rhea C. Alonzi[1], Daniel M. Jones[3], Roxana Filip[2], Rodney S. Russell[3], and John P. Pezacki[1,2]

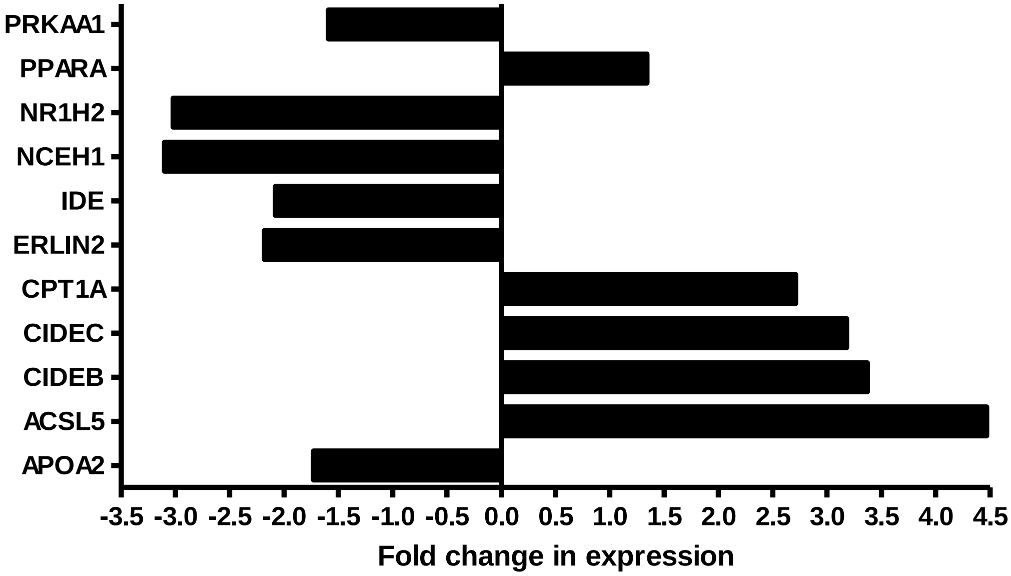

Supplementary Figure S1. Gene expression analysis in miR-7 transfected mimic Huh7.5 cells.
Fold changes in gene expression of select lipid-metabolism related genes in Huh7.5 transfected with 100 nM of miR-7 mimic are shown. Fold changes were calculated relative to gene expression in 100 nM control mimic-transfected Huh7.5 cells.

a

miR-7 U**GGAAGAC**UAGUGUAUUUUGUUGU

b

PRKAA1 – 2 miR-7 binding sites

3’UTR position 448-454

. . . **AAAU**–AGCUAGUA**UCUUCCA**G . . .

3’UTR position 479-485

. . . CU**UCUUCCA**UA . . .

c

ERLIN2 – 1 miR-7 binding site
3’UTR position 97-104

. . . CU**GUCUUCCA**GU . . .

d

NR1H2 – miR-7 binding site
3’UTR position 178-185

. . . AGCGA**GUCUUCC**–**A**GA . . .

e

PCSK5 – 1 miR-7 binding site
3’UTR position 1786-1792

. . . GCA**GU---CUUCC**CCU . . .

f

APOA2 – 1 miR-7 binding site
3’UTR position 102-109

. . . UU**GUCUUCCA**A . . .

g

IDE – 2 miR-7 binding sites
3’UTR position 44-51

. . . UGA**GUCUUCCA**GAG . . .

3’UTR position 1867-1874

. . . GUA**GUCUUCCA**UGA . . .

h

NCEH1 – 2 miR-7 binding sites
3’UTR position 345-351

. . . GCAGU**UCUUCCA**GCUC . . .

3’UTR position 1950-1956

. . . GUC**UCUUCCA** . . .

Supplementary Figure S2. miRNA recognition elements in direct targets of miR-7.
(a) Sequence of mature miR-7 with seed sequence highlighted in yellow. Sequence of miR-7 binding sites in the 3’UTRs of (b) PRKAA1, (c) ERLIN2, (d) NR1H2, (e) PCSK5, (f) APOA2, (g) IDE, and (h) NCEH1. Sequences were taken from TargetScan. Seed sequences are highlighted in yellow, and nucleotides involved in supplementary interactions with the 3’ end of miR-7 are highlighted in green.

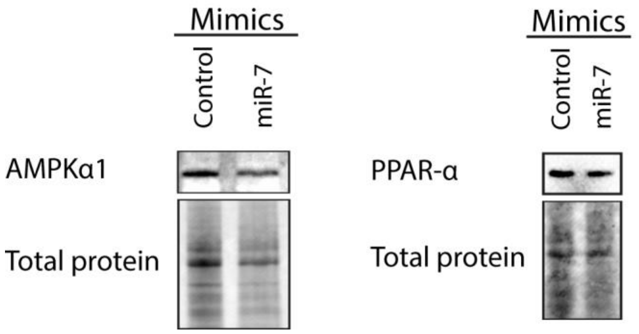

Supplementary Figure S3. Influence of miR-7 on AMPKα1 and PPAR-α protein levels.
Immunoblot analysis of AMPKα1 and PPAR-α protein levels in control and miR-7 mimic transfected Huh7.5 cells. Total protein is shown as a loading control. Full uncropped blots shown in Supplementary Figure S5b.

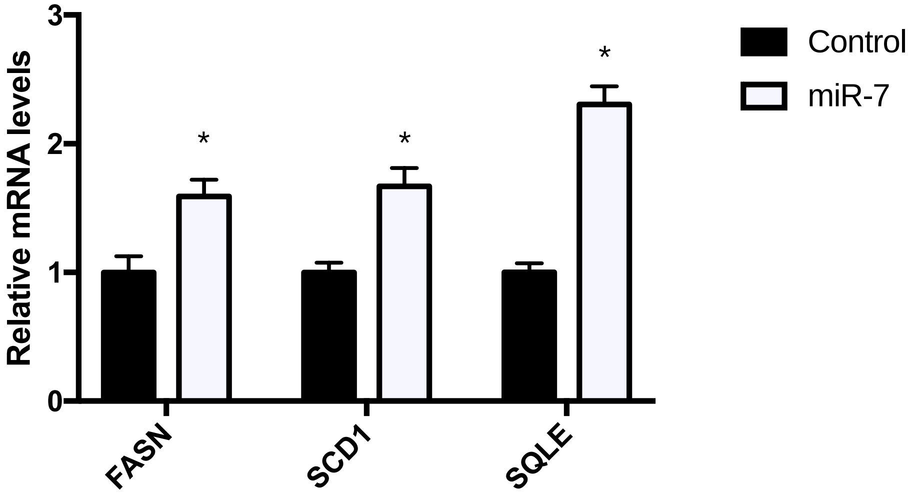

Supplementary Figure S4. miR-7 activates expression of SREBP-regulated genes.
qRT-PCR analysis of relative mRNA expression levels of SREBP-regulated genes (SCD1, FASN, and SQLE) in control and miR-7 mimic transfected Huh7.5 cells (n = 3; *$P \leq 0.05$).

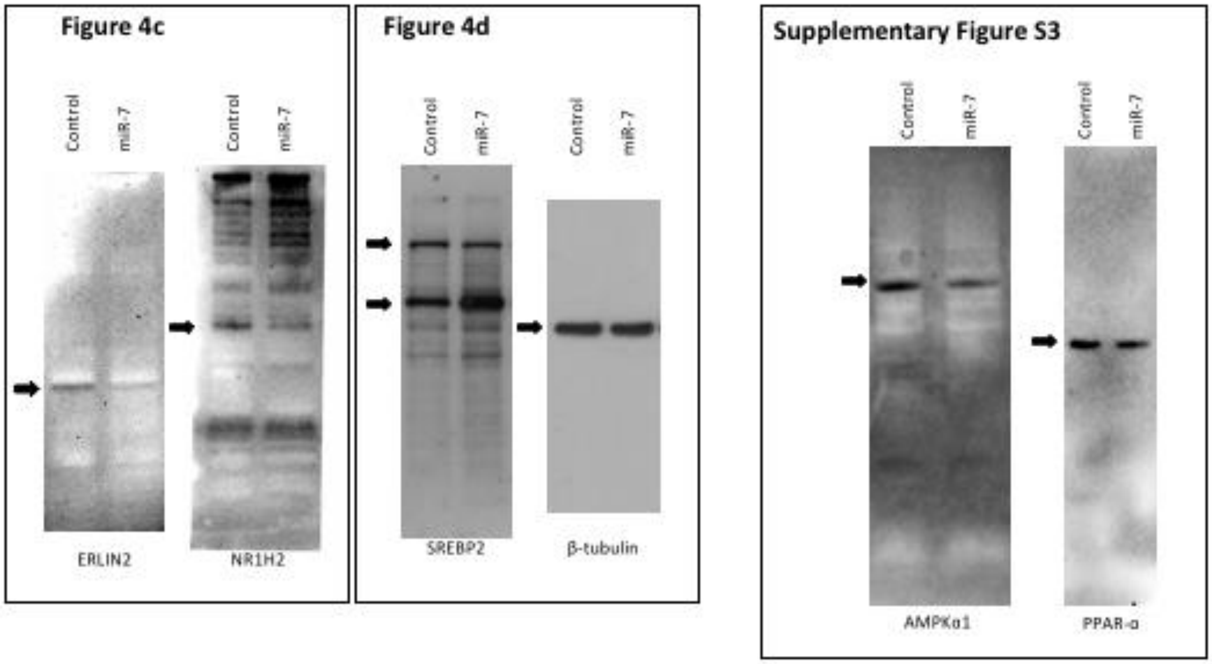

Supplementary Figure S5. Full blots from Figures 4c and 4d, and Supplementary Figures S3. Arrow highlight bands of interest.

Supplementary Table S1. Summary of top up- and down-regulated genes from microarray analysis of miR-7 mimic transfected Huh7.5 cells.

Gene Symbol	Description	Fold Change
	Top down-regulated genes	
EXOSC2	exosome component 2	-4.99
TPGS2	tubulin polyglutamylase complex subunit 2	-5.21
DUSP6	dual specificity phosphatase 6	-5.33
GINS1	GINS complex subunit 1 (Psf1 homolog)	-5.36
RCC2	regulator of chromosome condensation 2	-5.63
DUSP5	dual specificity phosphatase 5	-5.91
ETV4	ets variant 4	-6.09
CDCA7	cell division cycle associated 7	-6.32
DKK1	dickkopf WNT signaling pathway inhibitor 1	-6.46
CRTAP	cartilage associated protein	-8.68
	Top up-regulated genes	
ANKRD1	ankyrin repeat domain 1 (cardiac muscle)	25.8
DIO1	deiodinase, iodothyronine, type I	19.46
SYT11	synaptotagmin XI	13.74
HRG	histidine-rich glycoprotein	13.34
FMO1	flavin containing monooxygenase 1	10.88
MT1F	metallothionein 1F	10.45
SUSD2	sushi domain containing 2	10.04
VAT1L	vesicle amine transport 1-like	8.54
SAXO2	stabilizer of axonemal microtubules 2	7.52
ECM2	extracellular matrix protein 2, female organ and adipocyte specific	7.15

Supplemental Table S2. Gene ontology analysis classifying genes activated by >1.5 fold in miR-7 mimic transfected Huh7.5 cells by biological process.

ID	Name*	*P*-value†
GO:0044282	small molecule catabolic process	3.17E-7
GO:0016054	organic acid catabolic process	3.26E-6
GO:0046395	carboxylic acid catabolic process	3.26E-6
GO:0072329	monocarboxylic acid catabolic process	4.40E-4
GO:0015711	organic anion transport	5.50E-4
GO:0043436	oxoacid metabolic process	6.62E-4
GO:0019752	carboxylic acid metabolic process	7.55E-4
GO:0006082	organic acid metabolic process	9.52E-4
**GO:0044242**	**cellular lipid catabolic process**	**2.71E-3**
GO:0032787	monocarboxylic acid metabolic process	3.03E-3
GO:0042073	intraciliary transport	4.32E-3
GO:0046942	carboxylic acid transport	5.12E-3
GO:0015849	organic acid transport	6.30E-3
GO:0006820	anion transport	8.03E-3
**GO:0008203**	**cholesterol metabolic process**	**1.10E-2**
**GO:0008202**	**steroid metabolic process**	**1.32E-2**
GO:0030705	cytoskeleton-dependent intracellular transport	1.41E-2
**GO:0016125**	**sterol metabolic process**	**2.62E-2**
GO:0060271	cilium morphogenesis	2.96E-2
GO:0010970	microtubule-based transport	3.50E-2
GO:0010927	cellular component assembly involved in morphogenesis	4.04E-2
**GO:0016042**	**lipid catabolic process**	**4.30E-2**
GO:0044282	small molecule catabolic process	3.17E-7
GO:0016054	organic acid catabolic process	3.26E-6
GO:0046395	carboxylic acid catabolic process	3.26E-6

*Only biological processes with $P < 0.05$ are listed. Pathways related to lipid metabolism are highlighted in bold

†Adjusted with Bonferroni correction.

Supplementary Table S3. Summary of GSEA results with FDR < 0.05 – KEGG pathways enriched in miR-7 transfected cells.

ID	Name*	FDR q value
1	KEGG_COMPLEMENT_AND_COAGULATION_CASCADES	<0.001
**2**	**KEGG_PPAR_SIGNALING_PATHWAY**	**<0.001**
3	KEGG_DRUG_METABOLISM_CYTOCHROME_P450	0.002
4	KEGG_LYSOSOME	0.006
5	KEGG_OTHER_GLYCAN_DEGRADATION	0.009
6	KEGG_ABC_TRANSPORTERS	0.017
7	KEGG_PRIMARY_BILE_ACID_BIOSYNTHESIS	0.018
**8**	**KEGG_PEROXISOME**	**0.034**
9	KEGG_GLYCINE_SERINE_AND_THREONINE_METABOLISM	0.037
10	KEGG_RETINOL_METABOLISM	0.040
**11**	**KEGG_FATTY_ACID_METABOLISM**	**0.040**

*****KEGG pathways related to lipid metabolism are highlighted in bold

Supplementary Table S4. List of oligonucleotides used in this study.

Oligonucleotide	Sequence
qPCR primers	
18S rRNA – FWD	GCGATGCGGCGGCGTTATTC
18S rRNA – REV	CAATCTGTCAATCCTGTCCGTGTCC
CIDEB – FWD	GACCTCTTTGGCAGCCTGAATG
CIDEB – REV	AGTGTGGAGGTCCAACGAAGGA
NCEH1 – FWD	GCCGCCTATTACGTCTACATCC
NCEH1 – REV	TGATGGCTCAGTCCCAGGTAGT
CIDEC – FWD	AAGCGTGAGGAAGGGCATCATG
CIDEC – REV	CAGTTGTGCCATCTTCCTCCAG
ACSL5 – FWD	CTCAACCCGTCTTACCTCTTCT
ACSL5 – REV	GCAGCAACTTGTTAGGTCATTG
CPT1A – FWD	GATCCTGGACAATACCTCGGAG
CPT1A – REV	CTCCACAGCATCAAGAGACTGC
PPARA – FWD	CTATCATTTGCTGTGGAGATCG
PPARA – REV	AAGATATCGTCCGGGTGGTT
NR1H2 – FWD	CTTCGCTAAGCAAGTGCCTGGT
NR1H2 – REV	CACTCTGTCTCGTGGTTGTAGC
APOA2 – FWD	CTGTGCTACTCCTCACCATCT
APOA2 – REV	CTCTCCACACATGGCTCCTTT
IDE – FWD	TTTTCAGCCCATTTGCTTATGTG
IDE – REV	TGCATACTCGTTGAGTGAGTCTT
PCSK5 – FWD	TGTGGAGAGCACAGACCGACAA
PCSK5 – REV	ACAACGACGTGCTCCAGGTAGT
FASN - FWD	GAAACTGCAGGAGCTGTC
FASN- REV	CACGGAGTTGAGGCGCAT
SCD1 - FWD	CCGGGAGAATATCCTGGTTT
SCD1 - REV	GCGGTACTCACTGGCAGAGT
SQLE - FWD	GCGTGCTTGGTCTCTGCTTT
SQLE - REV	CCTGGGCATCAAGACCTTCCA
Site-directed mutagenesis primers	
NR1H2 miR-7 binding site SDM F	CCCTGCCCAGCGAGTCAACCAGAAGGGGTGAAAGG
NR1H2 miR-7 binding site SDM R	CCTTTCACCCCTTCTGGTTGACTCGCTGGGCAGGG
